# Morphological and Functional Correlations in Acute Central Serous Chorioretinopathy

**DOI:** 10.1007/s10633-024-09969-8

**Published:** 2024-03-18

**Authors:** Peter Kiraly, Maja Šuštar Habjan, Jaka Smrekar, Polona Jaki Mekjavić

**Affiliations:** 1 Oxford Eye Hospital, Oxford University Hospitals NHS Foundation Trust, Oxford, OX3 9DU UK; 2 Nuffield Laboratory of Ophthalmology, University of Oxford, Oxford, OX3 9DU UK; 3 Faculty of Medicine, University of Ljubljana, 1000 Ljubljana, Slovenia; 4 Eye Hospital, University Medical Centre Ljubljana, 1000 Ljubljana, Slovenia; 5 Faculty of Mathematics and Physics, University of Ljubljana, 1000 Ljubljana, Slovenia; 6 Jožef Stefan Institute, 1000 Ljubljana, Slovenia

**Keywords:** Central serous chorioretinopathy, CSC, CSR, Correlations, Morphological correlations, Functional correlations

## Abstract

**Purpose:**

We evaluate morphological and functional correlations in patients with acute central serous chorioretinopathy (CSC).

**Methods:**

A prospective study was conducted on 50 patients with an acute CSC episode lasting less than 3 months. At baseline, assessments included optical coherence tomography (OCT), best-corrected visual acuity (BCVA), contrast sensitivity (CS), microperimetry (MP), and multifocal electroretinography (mfERG). A correlation analysis between OCT morphological parameters (maximal subretinal fluid height (SRF), central retinal thickness (CRT), and macular volume (MV)) and functional parameters was conducted on the affected eye for each patient.

**Results:**

Among the morphological parameters, SRF showed the strongest correlations with functional parameters (*r* absolute value range = 0.10–0.70). Weak correlations were observed between BCVA and morphological parameters (*r* absolute value range = 0.14–0.26). Average retinal sensitivity (MP-A) was the functional parameter displaying the most robust negative correlation with morphological parameters (*r* absolute value range = 0.61–0.70). In contrast, average contrast sensitivity (CS-A) and mfERG average amplitude density in the first (mfERG-A1) and second (mfERG-A2) ring showed weak to moderate (*r* absolute value range = 0.35–0.56) yet statistically significantly nonzero correlations.

**Conclusions:**

SRF and CRT could serve as the most representative morphological proxies for visual function deficit in acute CSC patients. Retinal sensitivity, as measured by MP, may be superior to BCVA in clinical research studies or when an in-depth visual function evaluation is needed.

## Introduction

Central serous chorioretinopathy (CSC) is the fourth most common non-surgical maculopathy, and it is associated with pachychoroid, irregularities in the retinal pigment epithelium (RPE), and the accumulation of subretinal fluid (SRF) [1, 2]. The latest theory on the pathophysiological processes in CSC describes an increase in choroidal venous pressure, leading to the formation of intervortex anastomoses within the macula. This leads to choroidal hyperpermeability and increased interstitial pressure that ultimately results in leakage of fluid through the RPE [3]. CSC usually affects the middle-aged working population and is associated with several risk factors including excessive endogenous or exogenous corticosteroids [1].

In acute CSC, best-corrected visual acuity (BCVA) is usually only mildly impaired [1, 4, 5]. However, more precise functional tests, such as contrast sensitivity (CS), microperimetry (MP), and multifocal electroretinography (mfERG), often reveal more significant visual function impairment [6–9]. In patients with spontaneous SRF resolution within the first three months, all functional tests improve shortly after SRF resolution, with the exception of CS, which shows a gradual and delayed improvement [5]. A study showed that the maximal SRF height, a parameter which has to be manually measured with optical coherence tomography (OCT), exhibits a strong correlation with visual function parameters and subjective visual function impairment [4].

In acute CSC, where neurosensory retinal morphology is not significantly altered and RPE irregularities are limited to PED [1], visual function impairment occurs within the area of SRF accumulation [5]. In chronic CSC, characterized by the persistence of SRF for at least 6 months, irreversible vision function loss occurs and could lead to legal blindness [10]. A morphological and functional correlation study showed that serous retinal detachment height at the fovea did not correlate with BCVA in chronic CSC, where maximal neurosensory detachment is typically shallower when compared to acute CSC [1, 11]. On the other hand, studies have revealed that in chronic CSC, retinal sensitivity and mfERG parameters correlated negatively with inner/outer segments (IS/OS) line defects [12] and that retinal sensitivity is negatively correlated with RPE changes as observed on fundus autofluorescence (FAF) [13]. Hence, visual function impairment in chronic CSC primarily arises from retinal morphology alterations in the RPE and the outer retinal layers [11].

The aim of this study was to investigate visual function impairment in acute CSC by employing several visual function tests, including BCVA, CS, MP, and mfERG. Furthermore, our objective was to identify which frequently employed OCT morphological parameters (maximal subretinal fluid height (SRF), central retinal thickness (CRT), and macular volume (MV)) best reflect visual function impairment through the assessment of correlations between morphological and functional parameters.

## Methods

A prospective study was conducted at the University Eye Clinic in Ljubljana from 2018 to 2021. The study was conducted in accordance with the Declaration of Helsinki and approved by the National Medical Ethics Committee of the Republic of Slovenia (protocol code 0120–141/2018/4). Patients with CSC who met specific inclusion and exclusion criteria and provided informed consent were enrolled. CSC was characterized by the accumulation of SRF in the fovea, with angiographic evidence of leakage on fluorescein angiography (FA) and indocyanine green angiography (ICGA). Inclusion criteria required patients to have an acute CSC episode lasting less than 3 months and to be aged 18 to 65 years. Exclusion criteria ruled out individuals with other macular conditions besides CSC, ocular pathologies that could influence visual function parameters and allergy to fluorescein. Patients underwent a comprehensive ophthalmological examination at baseline, which included slit lamp examination, fundoscopy, and on the same day, they also underwent OCT, FA, ICGA, BCVA, CS, MP, and mfERG assessments.

Multimodal imaging was conducted using the Spectralis ophthalmic imaging platform (Heidelberg Engineering, Inc., Heidelberg, Germany). OCT of the macula was performed with a 30° field of view lens. The imaging platform software automatically determined the CRT within a circular region of 1-mm diameter centered on the fovea and the MV within a region of 3-mm diameter. A retina specialist manually measured the maximal height of SRF within the central 3-mm diameter around the fovea.

BCVA was assessed using the ETDRS chart (Precision Vision, Illinois, USA), with results converted to logMAR values [14]. CS was evaluated using the FACT chart (Stereooptical CO, Illinois, USA) at spatial frequencies of 1.5, 3.0, 6.0, 12.0, and 18.0 cycles per degree (cpd) [15]. The average CS was then calculated and expressed as log contrast sensitivity. Both BCVA and CS measurements were conducted following the standard protocol, with assessments carried out in the affected eye only.

Retinal sensitivity assessment was performed using MP (Nidek Technologies, MP1, 2002, Padua). A 1° red cross was utilized as a fixation target. A radial grid pattern was used to evaluate retinal sensitivity across 45 test points within a 12° area surrounding the foveola [16, 17]. The test employed a 4–2 staircase strategy, with a stimulus projection time of 200 ms and a stimulus size equivalent to Goldmann III [17]. The average retinal sensitivity (MP-A) was derived from all the tested points. Retinal sensitivity within the central 4° surrounding the foveola was assessed using the 13 central test points (MP-C). Paracentral retinal sensitivity, spanning from 4° to 12° away from the foveola, was evaluated based on 32 test points (MP-P).

MfERG measurements were performed following the standards set by the International Society for Clinical Electrophysiology of Vision (ISCEV) [18]. The HK electrode was positioned in the lower conjunctival sac [19], a reference electrode was placed just behind the temporal orbital bone rim, and the grounding electrode was situated on the glabella. A cathode-ray tube (CRT) screen (RETI port, Roland Consult, Germany) was utilized to display the stimulus. The stimulus, designed to elicit responses from the 30° area around the foveola, consisted of alternating black and white hexagonal fields. Stimulation cycles with a duration of 50 s were recorded and repeated eight times to calculate mean amplitude densities and implicit times for the first through fifth rings [20].

To evaluate correlations between morphological and functional parameters in a single eye, Pearson correlation coefficient was used. Statistical analysis was performed using the IBM SPSS Statistics for Windows, version 28 (IBM Corp., Armonk, NY, USA), with a cutoff at *p* < 0.05 for statistical significance.

## Results

Out of 50 patients, the average age was 44.7 (± 9.9), with 43 males and seven females included. The average duration of symptoms at the time of presentation was 1.4 (± 1.3) months. Five patients reported the use of exogenous corticosteroids in various forms (oral, nasal, dermal) at baseline. One patient used oral steroids for systemic lupus erythematosus, two patients used nasal steroids for allergic rhinitis, and two other patients used dermal steroids for psoriasis.

At baseline, CRT, MV, and SRF had average values (± SD) of 453 (± 133) µm, 3.04 (± 0.71) mm^3^, and 216 (± 144) µm, respectively. The BCVA was on average 0.19 (± 0.15); the CS 1.5 cpd, 3.0 cpd, 6.0 cpd, 12.0 cpd, 18.0 cpd, and the overall CS averages were 1.43 (± 0.24), 1.48 (± 0.29), 1.16 (± 0.59), 0.57 (± 0.63), 0.20 (± 0.35), and 0.97 (± 0.33), respectively. Retinal sensitivity averaged 9.0 (± 5.3) dB in the central region (MP-C), 12.6 (± 5.2) dB in the paracentral region (MP-P), and 11.6 (± 5.1) dB overall (MP-A). For multifocal ERG amplitude measurements, the average values from first to fifth ring were 58.7 (± 22.5) nV/deg^2^, 37.7 (± 11.3) nV/deg^2^, 25.4 (± 5.1) nV/deg^2^, 17.2 (± 3.1) nV/deg^2^ and 13.2 (± 2.7) nV/deg^2^, respectively. Latency measurements in mfERG from first to fifth ring averaged 42.3 (± 5.3) ms, 39.3 (± 1.9) ms, 37.4 (± 1.0) ms, 36.9 (± 1.0) ms, and 37.4 (± 1.1) ms, respectively. Comparison of functional parameters for healthy subjects and our acute CSC cohort is presented in Table 1. Functional parameters in healthy subjects were sourced from the available literature. BCVA in healthy eyes was obtained from 19 patients aged 45–49 [21]; CS in healthy eyes was measured in 27 patients with an average age of 38.8 [22]; MP-A measurements in normal controls were obtained from 33 patients aged 40–49 [16]; mfERG normative values from the University Eye Clinic in Ljubljana were obtained from 20 healthy individuals, which were not age matched to our CSC cohort.

**Table 1:** Average functional parameter values in normal subjects and in our cohort of acute CSC patients

	Normal subjects	Acute CSC patients
BCVA, x̅ (SD), logMAR	− 0.12 (0.05)	0.19 (0.15)
CS-1.5, x̅ (SD), log CS	1.94 (0.12)	1.43 (0.24)
CS-3.0, x̅ (SD), log CS	2.11 (0.11)	1.48 (0.29)
CS-6.0, x̅ (SD), log CS	2.12 (0.24)	1.16 (0.59)
CS-12.0, x̅ (SD), log CS	1.43 (0.24)	0.57 (0.63)
CS-18.0, x̅ (SD), log CS	1.43 (0.24)	0.20 (0.35)
MP-A, x̅ (range; SD), dB	19.2 (19.0–19.3)	11.6 (5.1)
mfERG-A1, x̅ (SD), nV/deg^2^	122.9 (24.2)	58.7 (22.5)
mfERG-A2, x̅ (SD), nV/deg^2^	57.5 (13.7)	37.7 (11.3)
mfERG-A3, x̅ (SD), nV/deg^2^	34.4 (7.2)	25.4 (5.1)
mfERG-A4, x̅ (SD), nV/deg^2^	20.8 (5.3)	17.2 (3.1)
mfERG-A5, x̅ (SD), nV/deg^2^	15.9 (4.7)	13.2 (2.7)

Central serous chorioretinopathy (CSC); Best corrected visual acuity (BCVA); Contrast sensitivity in 1.5 cycles per degree (cpd) (CS-1.5), 3.0 cpd (CS-3.0), 6.0 cpd (CS-6.0), 12.0 cpd (CS-12.0), 18.0 cpd (CS-18.0); Average retinal sensitivity (MP-A); Amplitude density in mfERG in 1st ring (mfERG-A1), 2nd ring (mfERG-A2), 3rd ring (mfERG-A3), 4th ring (mfERG-A4), 5th ring (mfERG-A5); Average (x̅); Standard deviation (SD); Reference for BCVA in normal subjects [21]; for CS in normal subjects [22]; for MP-A in normal subjects [16]; mfERG normative values from the University Eye Clinic in Ljubljana were used

**Fig. 1:**
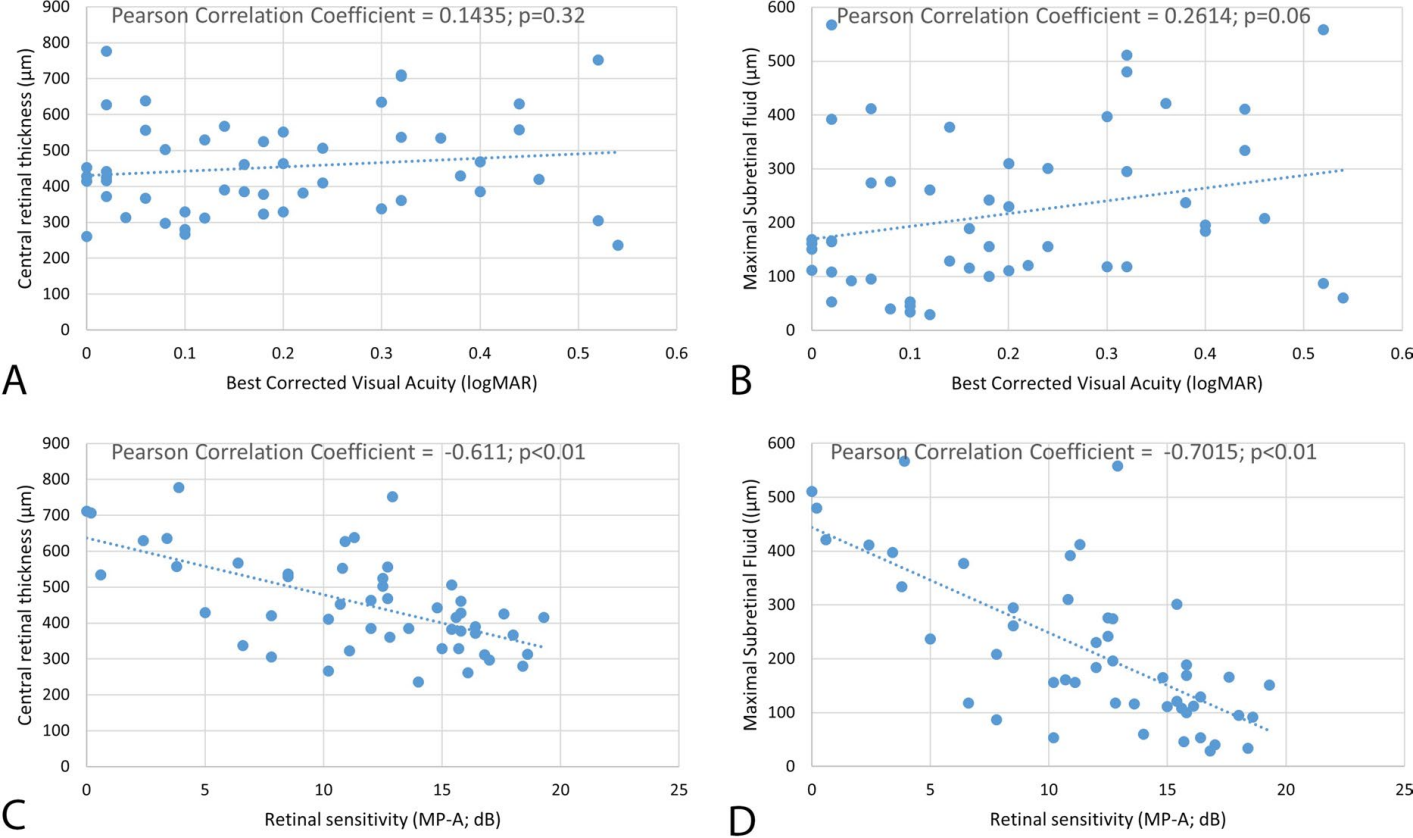
Correlation analysis between best-corrected visual acuity (BCVA) and central retinal thickness (CRT) (**A**); BCVA an maximal height of subretinal fluid (SRF) (**B**); average retinal sensitivity (MP-A) and CRT (**C**); MP-A and maximal height of SRF (**D**)

There was a weak correlation between BCVA and CRT (*p* = 0.32 when testing the null hypothesis of zero correlation) and BCVA and SRF (*p* = 0.06). On the other hand, correlation analysis showed strong negative correlation between MP-A and CRT (*r* = -0.61, *p* < 0.01) and MP-A and SRF (*r* = -0.70, *p* < 0.01) (Fig. 1). The correlation heatmap between morphological and functional parameters in our acute CSC patients can be seen in Table 2.

**Table 2:**
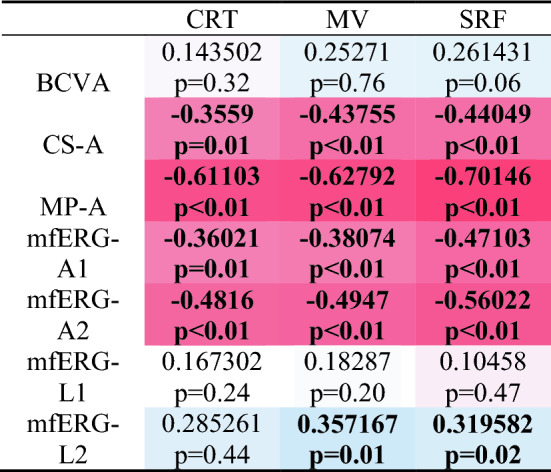
The correlation heatmap between morphological and functional parameters in our acute CSC patients

Central retinal thickness (CRT); Macular volume (MV); Maximal height of subretinal fluid (SRF); Best corrected visual acuity (BCVA); Average contrast sensitivity (CS-A); Average retinal sensitivity (MP-A); Amplitude density in mfERG in 1st ring (mfERG-A1), 2nd ring (mfERG-A2); Amplitude latency in mfERG in 1st ring (mfERG-L1), 2nd ring (mfERG-L2). Pearson correlation coefficients in bold indicate a correlation between two variables that is statistically significantly different from zero (p< 0.05). Blue indicates a positive correlation, while red indicates a negative correlation. The intensity of the color indicates the strenght of the correlation

**Fig. 2:**
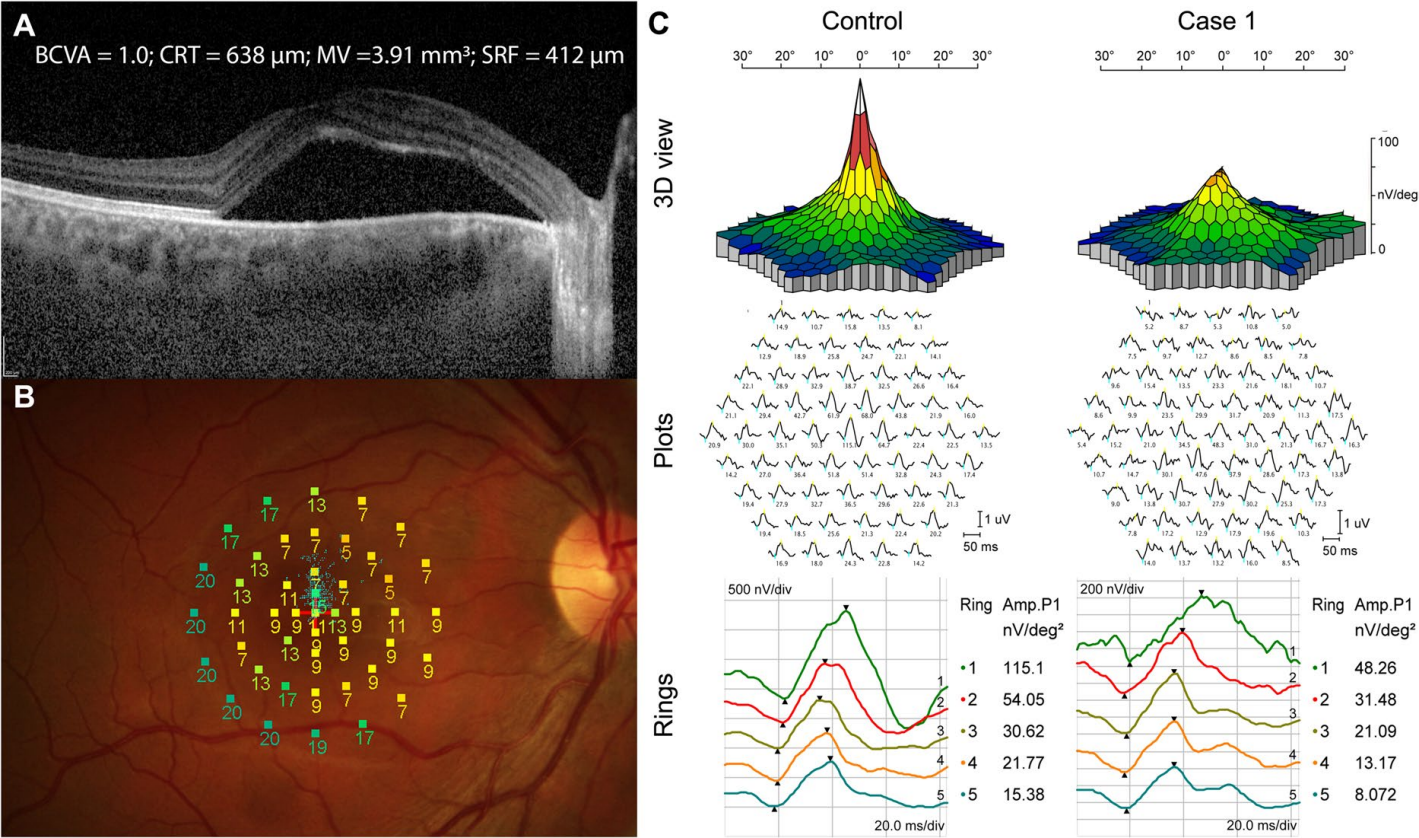
Acute central serous chorioretinopathy (CSC) patient with excellent best-corrected visual acuity (BCVA) (**A**) and severely reduced retinal sensitivity on microperimetry (**B**) and reduced amplitude densities on multifocal electroretinogram (**C**). Central retinal thickness (CRT); macular volume (MV); and subretinal fluid (SRF)

Figure 2 depicts a patient displaying significant SRF accumulation beneath the fovea. Despite this, the BCVA remained excellent (1.0; Snellen). Yet, when subjected to detailed functional tests like MP and mfERG, there was a marked decline in retinal sensitivity and amplitude densities.

## Discussion

Our study revealed that all visual function parameters in acute CSC patients were compromised. Among the morphological parameters, maximal SRF exhibited the strongest correlations with functional parameters. Weak correlations were observed between BCVA and morphological parameters. MP-A was the functional parameter that had the strongest negative correlation with morphological parameters. In contrast, CS-A, mfERG-A1, and mfERG-A2 displayed weak to moderate correlations that were statistically significantly different from zero.

When it comes to visual function loss, the mechanisms differ between acute and chronic CSC. In acute CSC, due to the retinal layer morphology being largely intact, visual function impairment is caused by the accumulation of SRF [4, 5, 7, 23]. In contrast, in chronic CSC, visual function loss predominantly arises due to the disruption of the outer retinal layers and RPE [11–13]. Rapid resolution of SRF in acute CSC is associated with the normalization of the majority of visual function parameters [5]. In contrast, chronic CSC can lead to irreversible vision loss and legal blindness [10].

In our study, very strong correlations were observed among all morphological parameters. SRF exhibited the strongest correlations between morphological and functional parameters. Gerendas et al. found a stronger correlation between SRF height and retinal sensitivity than between total SRF volume and retinal sensitivity [4]. Moreover, of all morphological parameters, subjective vision impairment handicap correlated most strongly with SRF height [4]. The maximal SRF height appears to be an important morphological parameter in CSC treatment, as patients with lower SRF demonstrated a good response to the subthreshold micropulse laser [24]. Although SRF height appears to be the most indicative morphological parameter for assessing visual function impairment and subjective handicap, it still requires manual measurement within the OCT software.

CRT is a widely used morphological parameter for monitoring the natural course and evaluating treatment effectiveness in all prevalent retinal maculopathies, including CSC [25]. It measures the retinal thickness in the central 1 mm around the fovea and is automatically generated by OCT software. Due to mostly preserved retinal structure in acute CSC, manual segmentation to obtain accurate measurements is usually not necessary. In our study, CRT correlated statistically significantly with CS-A, MP-A, mfERG-A1, and mfERG-A2. The strength of correlation with functional parameters was relatively similar to that with MV but weaker than with SRF. Therefore, due to the simplicity of obtaining measurements, it could be a valuable clinical parameter for evaluating vision impairment in acute CSC patients.

MV measures the macular volume in the 3 mm around the fovea. A larger MV at baseline has been shown to be associated with worse functional outcomes in acute CSC patients who experience rapid SRF resolution within 3 months [5]. Nevertheless, due to less prevalent use in clinical practice and weaker correlations with functional parameters, when compared to SRF, MV might not be the optimal morphological parameter to evaluate functional outcomes in acute CSC patients.

The distribution of SRF in acute CSC patients may play a significant role in visual function impairment. Patients with a broad and shallow accumulation of SRF might experience less visual impairment compared to those with a narrow and elevated SRF accumulation. In the choroid, the choriocapillaris is essential for delivering oxygen and nutrients to the cells of the outer retina [26]. The diffusion of oxygen and nutrients from the choriocapillaris to the outer retinal layers might be more hindered in patients with high SRF accumulation. This could lead to alterations in the outer retinal layers and poorer functional outcomes after the resolution of SRF.

Standard visual function tests evaluate elements like BCVA, CS, perceptions of color, depth, and motion. Each of these attributes represents an aspect of visual function that can influence an individual's overall visual capability [27]. BCVA, which represents the ability to recognize small details precisely, is the most commonly used visual function parameter. However, it only represents one aspect of visual function [27]. In our study, although BCVA was affected in CSC patients, it did not significantly correlate with any of the morphological parameters. Our cohort included only patients with acute CSC, none of whom had significant alterations in the outer retinal layers, such as IS/OS segment line defects or RPE alterations, known to correlate with BCVA worsening in longstanding CSC episodes [11–13]. Moreover, some of our patients exhibited excellent BCVA (1.0) yet had impairments in all other visual function tests. Figure 2 shows a patient with extensive SRF accumulation under the fovea; however, BCVA remained excellent (1.0). Nevertheless, more precise functional tests such as MP and mfERG showed severely reduced retinal sensitivity and amplitude densities. This clearly indicates that other functional tests are more sensitive and better determine visual function impairment than BCVA. CS provides a more accurate reflection of visual function in tasks such as face recognition, reading, and driving than BCVA [28]. In our study, CS at higher spatial frequencies were more affected, and average CS showed weak to moderate correlations with morphological parameters. Among the functional parameters, CS-A showed the strongest correlation with BCVA.

MP-A exhibited the strongest correlations with all the morphological parameters in our study. Microperimetry is widely used because it identifies minor changes in visual function that precede the worsening of visual acuity. As a result, it has become a prevalent functional parameter in clinical research studies to monitor the natural course of diseases and treatment effectiveness [29]. The usefulness of microperimetry might extend to biomarkers for spontaneous CSC resolution, with patients with retinal sensitivity of 20 dB or more being inclined toward spontaneous resolution [8]. Microperimetry offers several advantages for use in clinical practice or at least research trials for CSC patients. These include a strong correlation between MP-A and all other morphological and functional parameters, dynamic changes in measurements following morphological changes, high sensitivity to visual impairment, and a relatively quick and straightforward testing process.

In our study, mfERG amplitudes correlated moderately with morphological parameters, while latencies exhibited weak or very weak correlations. Amplitudes in the first two rings of our cohort fell below the normal range, whereas the more peripheral rings remained within the normal range, which is expected, because SRF in the majority of our patients did not extend beyond the second ring. This aligns with the findings of a study that showed mfERG impairment within the area of SRF accumulation [5]. Conversely, several studies have also indicated that mfERG measurements are affected beyond the area of SRF [23, 30, 31].

Although several studies have compared morphological and some functional parameters in CSC [4, 9, 11, 32], our study evaluated morphological parameters and several functional parameters (BCVA, CS, MP, mfERG) in a single study. We identified MP as the functional test that best correlates with morphological parameters in acute CSC and confirmed previous reports that maximal SRF height is most closely associated with visual function impairment [4]. Although conducting a wide range of functional tests in acute CSC patients is neither time nor cost-effective in a routine clinical practice, our study found that maximal SRF, which is easily measurable, offers the best correlation with visual function deficit. Additionally, MP may be the most sensitive and optimal functional test for use in clinical trials.

In conclusion, our study revealed that maximal height of SRF had the strongest correlations with functional parameters, while CRT exhibited moderate correlations. Therefore, these two morphological parameters might be used as a proxy for functional tests in clinical practice. Regarding visual function variables, BCVA showed very weak correlations with morphological parameters, while MP-A exhibited strong correlations. Therefore, in CSC clinical research studies, or when a detailed visual function evaluation is required, microperimetry might be superior to BCVA measurements.

## Data Availability

The datasets used and/or analyzed during the current study are available from the corresponding author on reasonable request.
